# Establishment of a humanized swine model for COVID-19

**DOI:** 10.1038/s41421-021-00313-x

**Published:** 2021-08-17

**Authors:** Xuguang Du, Zihang Guo, Wenhui Fan, Tang Hai, Fei Gao, Pan Li, Yumin Qin, Chaolei Chen, Zhiqiang Han, Jilong Ren, Pengtao Jiao, Wenjun Liu, Yuhai Bi, Dawei Yu, Sen Wu

**Affiliations:** 1 State Key Laboratory of Agrobiotechnology, College of Biological Sciences, China Agricultural University, Beijing, China; 2 Sanya Institute of China Agricultural University, Sanya, Hainan China; 3 CAS Key Laboratory of Pathogenic Microbiology and Immunology, Institute of Microbiology, Center for Influenza Research and Early-warning (CASCIRE), CAS-TWAS Center of Excellence for Emerging Infectious Diseases (CEEID), Chinese Academy of Sciences, Beijing, China; 4 State Key Laboratory of Stem Cell and Reproductive Biology, Institute of Zoology, Chinese Academy of Sciences, Beijing, China; 5 Institute for Stem Cell and Regenerative Medicine, Chinese Academy of Sciences, Beijing, China; 6 Beijing Institute for Stem Cell and Regenerative Medicine, Beijing, China; 7 Beijing Dhelixon Biotechnology Company Limited, Beijing, China; 8 Center for Biosafety Mega-Science, Chinese Academy of Sciences, Wuhan, Hubei China; 9 University of Chinese Academy of Sciences, Beijing, China

**Keywords:** Biological techniques, DNA recombination

Dear Editor,

Currently, wild-type (WT) hamsters, ferrets, cats, and non-human primates are being used as COVID-19 animal models. However, no severe clinical symptoms develop in these animals^1–5^. Similarly, most of the human ACE2 (hACE2) transgenic mouse models develop only mild COVID-19 disease with only a few recent transgenic mouse models developing severe and fatal respiratory diseases^6–10^, calling for a better large animal model that could mimic the full spectrum of COVID-19 symptoms. Although the pig is thought to be a better model for human diseases in general^11^, due to its similarity to human anatomy, physiology, and immunology, previous studies have shown that WT pigs are not susceptible to SARS-CoV-2^2,12^. Here we report our attempt to create the first humanized pig expressing the hACE2 receptor for COVID-19 research, speculating that humanization of the pig ACE2 receptor could make pigs susceptible to SARS-CoV-2.

To create a COVID-19 pig model with the targeted insertion of *hACE2* at the pig *ACE2* locus, we constructed a homologous recombination donor vector with homology arms of ~1 kb on each side. We used the CRISPR/Cas9 system to increase the chance of homologous recombination. Single-guide RNAs (sgRNAs) closest to the start codon of exon 1 were selected for the construction of sgRNA-expressing vector pX459 (Fig. 1a). To optimize the efficiency of the sgRNA, 9 sgRNAs were synthesized and assembled. IBRS-2 porcine kidney cells were electroporated with plasmids of *pACE2*-sgRNAs and Cas9. Sanger sequencing was used to identify the indels and evaluate the targeting efficiency for these sgRNAs. The cleavage efficiency of sgRNA3 was higher than that of other sgRNAs. Therefore, we chose sgRNA3 for subsequent experiments.

**Fig. 1:**
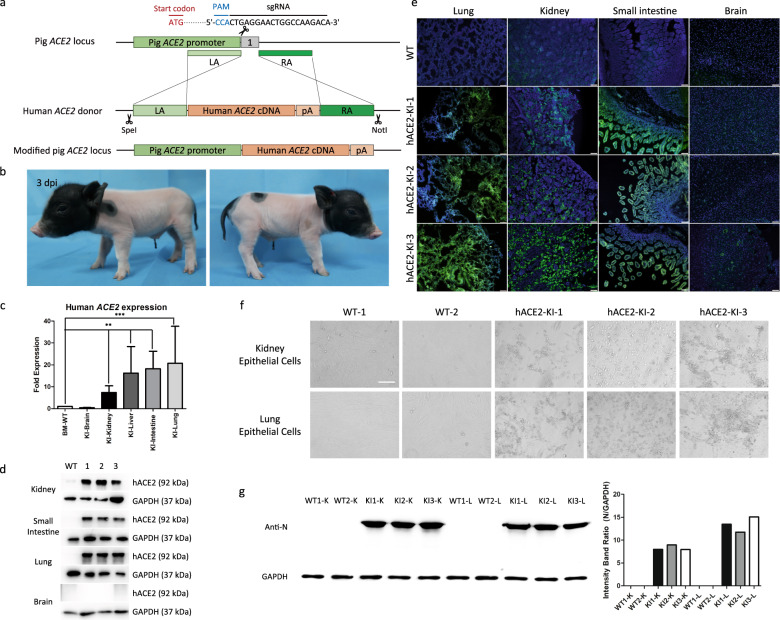
Generation and characterization of a site‑specific *hACE2* knock‑in pig model for COVID-19. **a** Scheme for *hACE2* integration in porcine fetal fibroblasts (PFFs) via CRISPR/Cas9-mediated homology-directed repair. **b** The physical appearance of representative cloned piglet. **c** Transcriptional analysis of *hACE2* in cloned pigs showing mRNA expression levels of *hACE2* in different tissues. Total RNA in different tissues of WT and knock-in pigs was extracted and subjected to reverse transcription, followed by qPCR to determine the expression of *hACE2*, with *GAPDH* as an internal reference gene. Data are presented as mean ± SD and are plotted from three independent experiments. **P < 0.01, ***P < 0.001, *n* = 3, unpaired one-tailed Student’s *t*-test. KI, knock-in. **d** Western blot showing expression of hACE2 protein in WT and knock-in (1, 2, 3) piglets. kDa, kilodalton. **e** Localization of hACE2 protein (green) in the lung, kidney, small intestine, and brain of WT and knock-in piglets indicated by immunofluorescent analysis. Nuclei are stained with DAPI (blue). Representative images were randomly chosen. Scale bars, 50 μm. KI, knock-in. **f** Primary epithelial cells of *hACE2* knock-in piglets showed significant cytopathic effects after SARS-CoV-2 infection. Scale bars, 50 μm. KI, knock-in. **g** Western blot analysis for the expression of virus N protein in primary epithelial cells derived from WT and knock-in piglets. K, kidney epithelial cells; L, lung epithelial cells.

Porcine fetal fibroblasts (PFFs) isolated from embryos of Bama mini-pigs were electroporated with plasmids of *pACE2*-sgRNA3, Cas9, and donor linearized by in vitro cleavage of the donor vector by restriction enzymes (*Spe*I and *Not*I). We used puromycin selection (1 μg/mL for 2 days) to enrich positive cell colonies, and their genotype was identified using PCR and Sanger sequencing (Supplementary Fig. S1a, b). Among the 85 single cell colonies, three of them (3/85, 3.53%) were identified as positive for *hACE2* insertion.

Next, the verified colonies were used as donor cells for somatic cell nuclear transfer (SCNT) into three surrogates. After about four months of pregnancy, these surrogates gave birth to nine genetically modified piglets (Fig. 1b). Genomic DNA was extracted from a variety of tissues of these piglets one day after birth, and their genotype was identified by PCR. The results showed that all of the piglets were positive for *hACE2* insertion (Supplementary Fig. S1a).

The transcriptional level of *hACE2* in several tissues of the knock-in piglets was quantified by qPCR, with *GAPDH* serving as the reference gene. The *hACE2* mRNA levels in the kidney, liver, small intestine, and lung of the knock-in pigs were significantly higher than their WT littermates. In contrast, only low mRNA levels of *hACE2* were detected in the brains of knock-in pigs (Fig. 1c).

To further determine whether the CRISPR/Cas9-mediated knock-in of *hACE2* could increase the expression of hACE2, the protein was isolated from different organs in the WT and knock-in pigs. Western blot showed high expression of hACE2 in the knock-in pigs in all tissues except the brain (Fig. 1d).

We also performed immunofluorescent analysis to detect hACE2 in different organs of both WT and knock-in pigs. The results showed a high expression of hACE2 in the lung, kidney and small intestine of knock-in pigs, based on a strong green fluorescent signal. In contrast, very faint signal was seen in WT tissues and the brain of knock-in pigs, which was consistent with the results identified by qPCR and western blotting (Fig. 1e).

To determine the replication efficiency of SARS-CoV-2 in primary cells of the *hACE2* knock-in pigs, lung and kidney epithelial cells were isolated and infected with SARS-CoV-2 (MOI = 0.01). After 72 h of infection, these primary cells of the *hACE2* knock-in pigs showed significant cytopathic effects in contrast to wide-type swine cells (Fig. 1f).

At 72 h post-inoculation (hpi), the cells were collected for the detection of SARS-CoV-2 N protein by western blotting (Fig. 1g) and immunofluorescence assay (IFA) (Supplementary Fig. S2). Obvious fluorescent signal was detected in the cells of the knock-in pigs compared to the cells from WT pigs. As shown by western blot analysis, significant expression of viral N protein was detected in the cells from the knock-in pigs, but not in the cells from WT pigs. These results indicated that the primary epithelial cells from the *hACE2* knock-in piglets are susceptible to SARS-CoV-2 infection.

In conclusion, we have shown that healthy *hACE2* knock-in pigs can be generated by targeted insertion of *hACE2* to the pig *ACE2* locus. The expression of hACE2 protein is regulated by the endogenous swine promoter and appears to recapitulate the in vivo expression pattern. The expression patterns of hACE2 in the lung, kidney, testis, and intestine of our hACE2 pig models are similar to human and the humanized ACE2 mice^8,13^. In humanized ACE2 mice, the small intestine showed higher levels of hACE2 than other tissues by qPCR. In contrast, in our humanized hACE2 pigs the lungs show higher hACE2 expression levels than other organs.

Since the *hACE2* knock-in pigs are created in this study by SCNT, they have a uniform genetic background and can be generated in large quantities in a short time. Also, severe cases of COVID-19 are often accompanied by underlying diseases, and pig models offer a unique opportunity to combine these existing diseases to reproduce severe cases of COVID-19. In addition to acute lung injury, some COVID-19 patients also experience organ damage, including acute kidney, heart, and liver dysfunction^14–16^, in particular for people with comorbidities such as hypertension, cardiovascular disease, and diabetes^15^. The humanized hACE2 pigs can be readily combined with other available pig models to accelerate the research of basic disease mechanisms, biomarkers, and treatment methods for high-risk people of COVID-19.

As SARS-CoV-2 mutants are rapidly emerging, scientists are scrambling to design and develop new vaccines, and are making continuous efforts to find antiviral drugs. The immediate urgency of treating severe disease and reining in viral escape variants calls for optimal animal models. Our *hACE2* knock-in pigs have great potential to fulfill this need.

## Supplementary information

Supplementary Figures and methods
