# Supplementary material for: Establishment of a humanized swine model for COVID-19
Source: Cell Discov. 2021 Aug 17;7:70. doi: 10.1038/s41421-021-00313-x (PMC8371120; doi:10.1038/s41421-021-00313-x)
Supplement: Supplementary file 1 — Supplementary Figures and methods [file 41421_2021_313_MOESM1_ESM.pdf]

Supplemental Information for

Establishment of a Humanized Swine Model for COVID-19

This PDF file includes:

Supplementary M&M

Supplementary Fig. S1

Supplementary Fig. S2

Supplementary information, Materials and Methods

Cells and Animals

Porcine fetal fibroblasts (PFFs) were grown at 37°C, in 5% CO_2 in Minimum Essential Medium α (MEM α; Thermo Fisher Scientific) supplemented with 15% fetal bovine serum (FBS; Thermo Fisher Scientific), 1 mM Sodium Pyruvate, 1×NEAA, 1% penicillin and streptomycin and 10 ng/ml rhFGF while IBRS2 cells were maintained in DMEM supplemented with 10% FBS. Animal experiments in this study were approved by the Animal Welfare Committee of China Agricultural University (SKLAB-2012-11).

Plasmids

The pX459 vector was used to express Cas9 and sgRNA targeting the pig ACE2 locus with the selectable marker gene puromycin. Nine sgRNAs used are as following:

Pig ACE2 sgRNA	sgRNA sequence (5'-3')	Indel efficiency in IBRS2 cells
sgRNA1	TGCTGCTCAGTCCACCACTG AGG	41.7%
sgRNA2	GGCGCACAGAGAAAGATGTC AGG	42.2%
sgRNA3	TGTCTTGGCCAGTTCCTCAG TGG	62.2%
sgRNA4	GAGCAGCAGTTACAGGAATG AGG	43.1%
sgRNA5	CTTGGCCAGTTCCTCAGTGG TGG	40.5%

sgRNA6	TAACCTTGAAGCCGAAGACC TGG	29.0%
sgRNA7	TCAGTCCACCACTGAGGAAC TGG	25.5%
sgRNA8	CGATGAGAATATCCAAAAGA TGG	25.7%
sgRNA9	GATATTCTCATCGGTAATAT TGG	13.4%

An efficient Kozak sequence (5'-GCCACC-3') was added upstream of the ATG start codon of the hACE2 cDNA in pPL332.1 vector, with a 5' homology arm (HA) of 997 bp and a 3' HA of 1 kb.

Transfection and Selection of IBRS2 Cells and PFFs

Cells were dissociated using 0.1% trypsin into single cells after washing in phosphate buffer saline and transfected using the 2B-Nucleofector Device (Lonza). For each transfection, 10^6 cells were mixed with 100 μL pre-warmed nucleofection reagents. The cell suspensions were then mixed with plasmids and electroporated with program A-024 (Lonza). Transfected cells underwent puromycin (1 μg/mL) selection for 2 days to ensure successful transfection.

Isolation of Genomic DNA

Cells were incubated in 500 μL of ES lysis buffer (100 mM NaCl; 20 mM Tris, pH 7.6; 10 mM EDTA; 0.5% SDS; 200 μg/mL Proteinase K) at 37°C for 2 h. While pig tissues were incubated in 500 μL of Tail lysis buffer (50 mM Tris, pH 8; 100 mM EDTA; 1% SDS; 100 mM NaCl;

400 μg/mL Proteinase K) at 55°C overnight. After 250 μL of saturated NaCl was added, tubes were hand shaken vigorously 100–200 times and left on ice for 10 min before centrifugation 20000 *g* for 10 min at 4°C. The supernatant was then transferred to a new tube and a 2-fold volume of 100% ethanol was added. The mixture was subjected to centrifugation at 13,000 *g* for 10 min. The supernatant was discarded, and the precipitate was washed with 70% ethanol. The tube was then centrifugated 13,000 *g* for another 10 min and the supernatant was removed. After air dry at 37°C for 10 min, the resulting DNA was resuspended in TE (pH 7.5).

Generation and Genotype Analysis of Cloned Piglets

Matured oocytes were enucleated by micromanipulation. Positive PFFs were injected into the perivitelline space, and fusion was accomplished using a BTX Electro-cell Manipulator 200 (BTX) with two direct current pulses (1-s interval) of 1.2 kV/cm for 30 μs in the fusion medium [0.3 M mannitol, 1.0 mM $CaCl_2$, 0.1 mM $MgCl_2$, and 0.5 mM Hepes (pH 7.0–7.4)]. Oocytes were then incubated for 30 min in PZM3, and the fusion percentage was calculated under a stereomicroscope. Fifty fused embryos were placed into a four-well dish (Nunc) containing 500 μL of PZM3 at 38.5°C and 5% CO_2 with maximum humidity. Day 1 NT zygotes were transferred surgically into surrogate mothers (250–300 zygotes per surrogate).

About 25 days later, the pregnancy status of the surrogates was diagnosed by ultrasonography.

Transcriptional Analysis of hACE2 Gene in knock-in Pigs Using qPCR

Total RNA from the hACE2 knock-in piglets and wild-type littermates was extracted using RaPure Total RNA Kit (Magen, R4011-03). The obtained RNA of 2 μg from different tissues was reverse transcribed into cDNA for the subsequent qPCR to measure the relative mRNA levels of hACE2, with GAPDH as the reference gene. Specific primers for hACE2 are hACE2-F3 (5'-GTTTTGAATAGCGCCCAACC-3') and hACE2-R3 (5'-TCTTGGCCTGTTCCTCAATG-3'), while primers for GAPDH are GAPDH-F (5'-ACCCAGAAGACTGTGGATGGC-3') and GAPDH-R (5'-AGCCAGAGGCAAAGTGATAGATA-3').

Western Blot Analysis

Total proteins were extracted from a variety of tissues of hACE2 knock-in piglets and their wild-type littermates with RIPA lysis buffer (Beyotime, Shanghai, China; P0013B). Ten μg protein was denatured at 99°C for 10 min in sample buffer, then separated by 6% SDS/PAGE and transferred onto polyvinylidene fluoride membrane (GE Healthcare, Little Chalfont, Buckinghamshire, UK). The anti-ACE2 primary antibody (Abcam, Cambridge, MA, USA;

ab108252) was used at 1:1000 dilution and the anti-GAPDH primary antibody (Beyotime; AF1186) was used at 1:1000. The SARS-CoV-2 (2019-nCoV) Nucleocapsid antibody (Sino Biological; 40143-R019) was used at 1:1000 dilution. The anti-rabbit secondary antibody (Beyotime; A0208) was used at the dilution of 1:1000. SuperSignal West-Dura Extended Duration Substrate kit (Thermo Fisher Scientific, Waltham, MA, USA; #34075) was used to develop the signal.

Frozen Sections and Immunofluorescent Analysis

The pig tissues were fixed using 4% formaldehyde phosphate-buffered saline (PBS) at 4°C overnight. After dehydration in 10% and 30% sucrose solutions consecutively, the tissues were embedded with optimal cutting temperature compound (Tissue-Tek, Sakura Finetek Inc., Tokyo, Japan) and frozen at -80°C. Twelve-micrometer slices were cut with microtome, air dried, and stored at -30°C until use.

Frozen sections were washed with PBS three times and blocked with 5% bovine serum albumin (BSA) for 1 h and incubated at 4°C overnight with primary antibodies: anti-hACE2 (1:100; Abcam, Cambridge, United Kingdom), SARS-CoV-2 (2019-nCoV) Nucleocapsid antibody (1:1000; Sino Biological). Secondary antibodies (1:250; Alexa 488, Thermo Fisher Scientific,

Waltham, MA, United States) (1:1000; Goat Anti-Rabbit IgG H&L (TRITC), Abcam; ab6718) were applied at room temperature for 1 h after PBS immersion. Nuclei were stained by neutral gum with DAPI (1:10000). Images were captured with a laser confocal microscope.

Isolation and Culture of Primary Swine Epithelial Cells

Kidney and lung tissues were collected and washed with PBS. Tissues were digested in 200 U/mL collagenase type I at 37°C for 15 min. After the digestion system was stopped and centrifuged, the supernatant was discarded. Cells were resuspended in DMEM supplemented with 15% FBS, 1% penicillin and streptomycin, and 10 ng/µL EGF. Then cells were plated onto 100-mm cell culture plates and incubated at 37°C and 5% CO_2.

Infection Experiment

Epithelial cells of kidney and lung of knock-in and wild-type piglets were isolated and seeded in cell culture flasks and 24-well culture plates under the condition of 37°C, in 5% CO_2 in DMEM (Thermo Fisher Scientific) supplemented with 10% fetal bovine serum (FBS; Thermo Fisher Scientific), 1% penicillin and streptomycin and 10 ng/ml rhFGF. The cells were infected with SARS-CoV-2 in BSL-3 labs when the confluence reached 80%. After washing twice with sterile PBS, the epithelial cells were inoculated with SARS-CoV-2 (hCoV-

19/China/CAS-B001R/2020) at a multiplicity of infection (MOI) of 0.01 based on 50% tissue culture infectious dose ($TCID_{50}$). At 72 hours post-inoculation (hpi), the cells and culture supernatant were harvested for Western-blot and immunofluorescence analyses.

Statistical analysis

*$P < 0.05$ ** $P < 0.01$, *** $P < 0.001$ was considered a significant difference in the two-sided test. All values represent means ±SEM.

Supplementary Figure S1. Genotyping of cloned piglets.

a. The results of the genomic PCR analysis confirmed the knock-in events of the hACE2 gene at the pig *ACE2* locus. RA: Amplification for the right arm using primers RA-F and RA-R; hACE2: Amplification for the hACE2 cDNA using primers hACE2-F1 and hACE2-R2; BM WT: Blank control; IBRS2 donor: Positive control; negative: Negative control; M: 1kb Plus DNA Ladder.

b. The sequence of the 3' junction site of the representative BM PFF clone integrated with the hACE2 cDNA.

Fig. S1

a

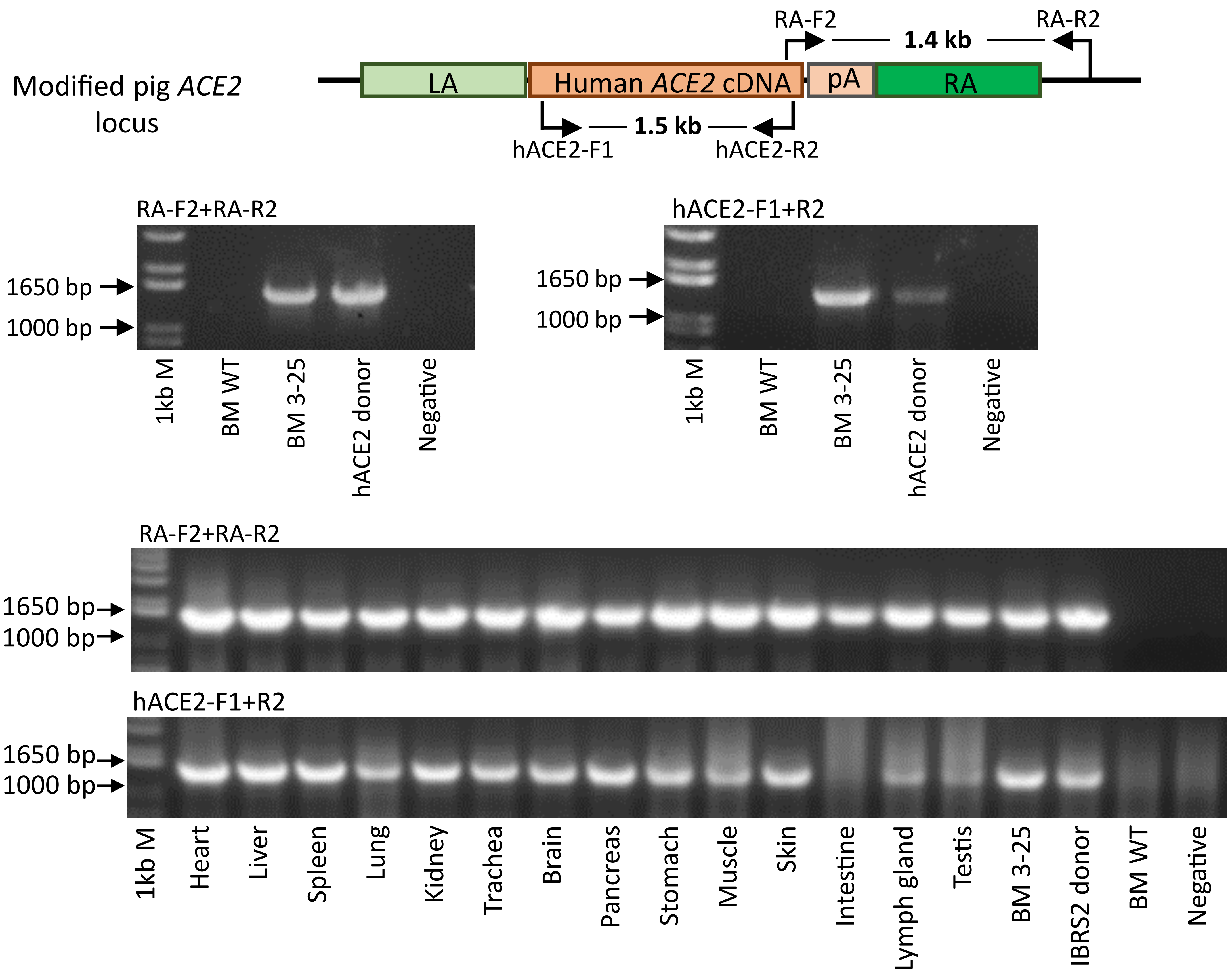

b

3' junction of #BM3-25 clone

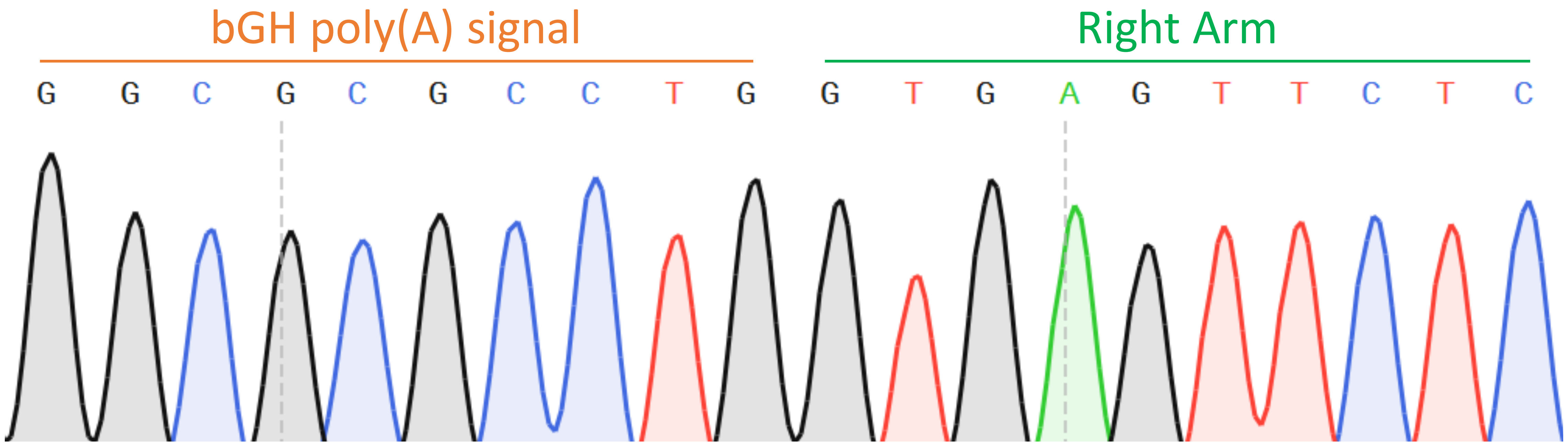

Supplementary Figure S2. Expression of virus N protein (red) in swine epithelial cells.

a. Staining for virus N protein (red) in kidney epithelial cells from hACE2 knock-in and wild-type piglets using IFA. Nuclei are stained with DAPI (blue). Representative images were randomly chosen. Scale bars, 25 μm.

b. Staining for virus N protein (red) in lung epithelial cells from hACE2 knock-in and wild-type piglets using IFA. Nuclei are stained with DAPI (blue). Representative images were randomly chosen. Scale bars, 25 μm.

Fig. S2

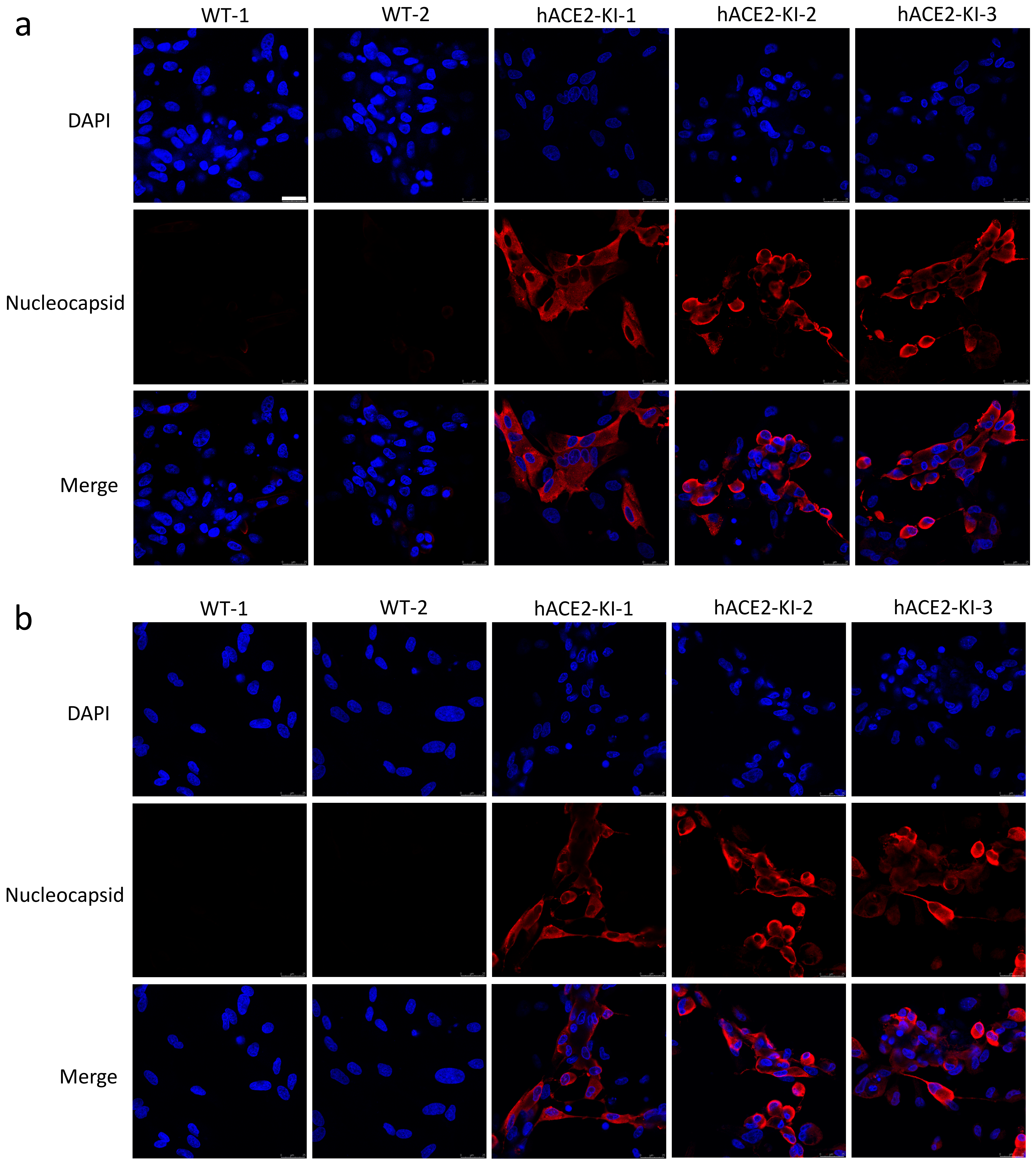
a
WT-1
WT-2
hACE2-KI-1
hACE2-KI-2
hACE2-KI-3
DAPI
Nucleocapsid
Merge
b
WT-1
WT-2
hACE2-KI-1
hACE2-KI-2
hACE2-KI-3
DAPI
Nucleocapsid
Merge
